# Changes in abortion service provision in Bihar and Jharkhand states, India between 2004 and 2013

**DOI:** 10.1371/journal.pone.0197300

**Published:** 2018-06-07

**Authors:** Andreea A. Creanga, Kaushalendra K. Singh, Qingfeng Li, Timothee Fruhauf, Amy O. Tsui

**Affiliations:** 1 Department of International Health, Johns Hopkins Bloomberg School of Public Health, Baltimore, MD, United States of America; 2 Department of Gynecology and Obstetrics, Johns Hopkins School of Medicine, Baltimore, MD, United States of America; 3 Banaras Hindu University, Department of Statistics, Varanasi, India; 4 Department of Population, Family and Reproductive Health, Johns Hopkins Bloomberg School of Public Health, Baltimore, MD, United States of America; 5 Bill and Melinda Gates Institute for Population and Reproductive Health, Johns Hopkins Bloomberg School of Public Health, Baltimore, MD, United States of America; 6 Johns Hopkins School of Medicine, Baltimore, MD, United States of America; National Academy of Medical Sciences, NEPAL

## Abstract

**Background:**

The Medical Termination of Pregnancy (MTP) Act of 1971 liberalized abortion laws in India. This study examines changes in abortion service provision and characteristics of abortion providers in Bihar and Jharkhand states, India between 2004 and 2013.

**Methods:**

We used state-representative data from cross-sectional surveys of reproductive health service providers we conducted in 2004 (N = 1,323) and 2012/2013 (N = 1,020). We employed chi-squared tests to examine and compare abortion providers’ characteristics, and fitted separate multivariate logistic regression models for provision of surgical, medical, and any abortion services, respectively, adjusting for potential confounders to identify factors associated with abortion service provision at the two survey time points.

**Results:**

Of providers interviewed in 2004 and 2012/2013, 63.7% and 84.5%, respectively, offered abortion services. Among abortion providers, 21.1% offered surgical and 10.7% offered medical abortions in 2004; 15.8% and 94.1% did so, respectively, in 2012/2013. Private providers were more likely than public providers to offer abortion services at both time points. Compared to female providers, male providers were significantly less likely to provide both surgical and medical abortions in 2004, and significantly less likely to provide surgical abortions in 2012/2013. Pharmacists and community health workers played increasingly important roles in abortion service provision, especially medical abortion, during the period.

**Conclusion:**

This study documents important changes in abortion provision in the two Indian states during 2004–2013.

## Introduction

In India, provision of abortion services is permitted at all public facilities with certified abortion providers, and at registered facilities in the private sector that are certified to offer abortions based on a set of government-set infrastructure and human resource criteria [1]. Only obstetrician-gynecologists and other allopathic physicians who have completed a bachelor of medicine/bachelor of surgery degree, have undergone government-approved training, and have received certification can legally provide abortions [1]. In practice, however, all types of providers are found offering abortion services, and medical abortion drugs can also be obtained from rural medical practitioners and from pharmacies [1, 2].

It was the Medical Termination of Pregnancy (MTP) Act of 1971 that liberalized abortion laws in India [3]. An abortion is currently permitted to save the life of the woman, preserve her physical and mental health, in case of rape or incest, fetal impairment, for economic or social reasons [3]. In order to expand safe abortion services, in 2002, the Government of India approved mifepristone coupled with misoprostol for early abortions up to 49 days’ gestation [4]. A 2003 amendment to the MTP Act enabled certified providers to prescribe medical abortion drugs outside a registered facility as long as emergency back-up facilities are available to them [1, 5]. National comprehensive abortion care guidelines were released in 2010 and indicated that medical abortion with mifepristone and misoprostol may be provided up to 63 days of gestation [2]. Notably, this indication has not yet been reflected in a change to the MTP Act, and neither were amendments to the MTP Act proposed in 2014 to allow mid-level providers and non-allopathic practitioners to terminate pregnancies and to expand the gestational age limit for abortion to 24 weeks [5,6].

The level of awareness about the legality of abortions appears to be low in India. Therefore, abortion seekers may attempt to induce abortion on their own, to obtain an abortion from an unauthorized provider, or to get oral abortion medications from a pharmacist without a prescription [7]. According to Indian government data, only about 1 million abortions are performed annually under the MTP Act, while the number of abortions performed outside the legal framework varies between 2 and 6 million per year [1,2]. For example, of the 6.4 million abortions estimated as having been performed in India in 2002 and 2003, 3.6 million were unsafe procedures [7]. Not surprisingly, induced abortions represent a major cause of maternal mortality and morbidity in the country. About 12,000 deaths result from abortion-related complications each year [1,8,9], and estimates of the contribution of unsafe abortions to maternal mortality vary between 9% and 20% [7,9,10].

Access to legal abortion services is particularly inadequate in Bihar and Jharkhand states, two of the least developed states in India [11], especially so for the 75% of the population living in rural areas [12]. About 10% of the country’s population lives in these two states, where only 1% of all certified abortion facilities are known to be located [7,13]. Hence, medical abortion offers great potential for improving access to safe abortion services in these two Indian states. This study examines changes in legal abortion service provision and characteristics of abortion providers in Bihar and Jharkhand states between 2004 and 2013. In addition, it assesses providers’ reasons for offering medical abortions or not in the two states at the same two time points.

## Materials and methods

### Data source

We use data from two cross-sectional surveys of facilities offering reproductive health services in Bihar and Jharkhand states, India. The aim of the first survey, conducted between January and June 2004, was to evaluate the performance of the Janani franchise network offering reproductive health services in the two states [14]. At that time, a multistage cluster sampling was applied to the entire two states with the exception of several southwest districts that were politically unsafe for fieldwork. Districts within the states’ regions were listed, and two of them were selected with probability proportional to size for each region. The districts were then divided into urban and rural strata, and further divided into blocks within urban strata and villages within rural strata. Within each block or village, all public and private health facilities were listed and mapped. Facility managers in 1,323 of the 1,346 government and private health facilities and pharmacies listed consented to participate in the survey. In each facility, all providers were enumerated and those authorized to offer reproductive health services were invited to participate in the survey; 2,039 providers consented to be interviewed (92.1% response rate). Between June 2012 and February 2013, we conducted a second survey using the same sampling strategy outlined above to specifically assess the provision of abortion services in the same districts in both states. Of note, some of the health facilities surveyed in 2004 had closed or relocated; thus, the 2012–2013 health facility sample included facilities surveyed in 2004 as well as facilities relocated or opened between July 2004 and June 2012. Managers in 1,020 of 1,095 facilities identified as offering reproductive health services consented to participate in the survey, and we interviewed one, self-selected provider in each facility (93.2% response rate). For this analysis, we pooled the data from the two states as Jharkhand was formed from Bihar in year 2000. Because only one provider was interviewed in each facility in 20012/2013, we randomly selected one of the providers interviewed in each facility in 2004. Thus, the 2004 and 2012–2013 analytic samples include 1,323 and 1,020 providers, respectively.

### Measures

We used standardized questionnaires for both surveys—the 2012/2013 survey questionnaire employed the same questions as the 2004 survey to collect data on providers’ socio-demographic and work-related characteristics, their training, knowledge of, attitudes toward, and practice of abortion services. Based on our a priori conceptual model [14], measures of interest for this analysis included: state (Bihar vs. Jharkhand); facility location (urban vs. rural); facility sector (public hospital/clinic, private hospital/clinic, pharmacy); whether the provider interviewed was the facility manager (yes/no); provider’s sex (male vs. female); age (<30, 30–34, 35–39, 40–44, ≥45 years); medical system and position (western medicine physician; traditional medicine physician; mid-level provider including nurses, auxiliary nurse midwives, clinic coordinators, lab technicians, and family planning counselors; pharmacist/pharmacy worker; and community health worker); experience as a health provider (<5, 5–9, 10–14, ≥15 years); whether working full-time in the facility where interviewed (yes/no); and number of weekly hours worked (<40, 40–46, ≥60).

### Analysis

We first used chi-squared tests to test compositional differences in the characteristics of providers offering: 1) surgical, 2) medical, 3) both surgical and medical abortions, 4) post-abortion care only, and 5) any type of abortion service, including abortion counseling and assistance with abortion provision rather than actual provision. To examine changes in legal abortion service provision and characteristics of abortion providers between 2004 and 2012/2013, separately on each of the two survey samples, we fitted logistic regression models for the provision of: 1) surgical abortion, 2) medical abortion, and 3) any type of abortion services, adjusting for all the characteristics noted above. Additionally, given expected changes in medical abortion provision between the two-time points, we assessed providers’ reasons for offering medical abortion or not using answers to seven direct questions about specific factors influencing providers’ decision to offer medical abortion and 13 direct questions about factors influencing their decision not to offer medical abortion. All data were weighted using Taylor’s linearization method.

The 2004 survey protocol was reviewed and approved by the Institutional Review Board at the University of North Carolina at Chapel Hill; the 2012/2013 survey was approved by Institutional Review Boards at the World Health Organization and the Banaras Hindu University. All analyses were adjusted for the complex design of the surveys using Taylor’s linearization method. Analyses were performed with Stata version 14.

## Results

About two thirds (63.7%) of reproductive health providers interviewed in 2004 offered abortion services, and this proportion increased to 84.5% among those interviewed in 2012/2013 (Table 1). Among abortion providers, 21.1% and 10.7% offered surgical and medical abortions, respectively, in 2004; 15.8% and 94.1% did so, respectively, in 2012/2013. In 2004, only 7.4% of abortion providers worked in the public sector compared to 16.2% in 2012/2013. Overall, only 16.5% of abortion providers in 2004 and 10.6% in 2012/2013 were facility managers. However, they represented 22.5% and 40.3% of surgical and medical abortion providers, respectively, in 2004 as well as 32.0% and 10.8% of surgical and medical abortions, respectively, in 2012/2013. Among surgical abortion providers, 46.5% in 2004 and 62.2% in 2012/2013 were physicians practicing western medicine; among medical abortion providers, a higher proportion were pharmacists or community health workers than physicians or mid-level professionals in 2012/2013 (67.6%) than in 2004 (19.9%). Considerably higher proportions of abortion providers in 2012/2013 than in 2004 were male (91.0% vs. 74.1%), had ≥10 years of experience (80.9% vs. 41.0%), and worked ≥40 hours per week (87.4% vs. 73.9%).

**Table 1:** Characteristics of abortion service providers in Bihar and Jharkhand, India: 2004 and 2012/2013.

Provider characteristics	2004 N = 1,323					2012/2013 N = 1,020				
	Surgical abortion	Medical abortion	Medical & surgical abortion	Post-abortion care only^1^	Any abortion service	Surgical abortion	Medical abortion	Medical & surgical abortion	Post-abortion care only^1^	Any abortion service
N (% of total providers interviewed)	178 (13.5)	90 (6.8)	55 (4.2)	174 (13.2)	843 (63.7)	136 (13.3)	811 (79.5)	128 (12.6)	25 (2.5)	862 (84.5)
State (%)										
Bihar	58.3	64.9	45.8	75.1	65.9	73.7	66.6	72.6	56.4	69.0
Jharkhand	41.7	35.1	54.2	24.9	34.1	26.3	33.5	27.4	43.6	31.0
Facility location (%)										
Urban	23.1	20.8	24.0	9.8	18.4	21.4	20.7	22.6	21.2	20.2
Rural	76.9	79.2	76.0	90.3	81.6	78.7	79.3	77.4	78.8	79.8
Facility sector (%)										
Public hospital/clinic	3.3	0.0	0.1	6.1	7.4	16.7	10.6	17.5	26.8	16.2
Private hospital/clinic	96.7	95.1	99.8	91.1	78.3	83.3	68.1	82.5	73.2	65.7
Private pharmacy^2^	0.0	4.9	0.1	2.9	14.3	0.0	21.4	0.0	0.0	18.2
Head of health facility (%)										
No	77.6	59.8	67.9	84.9	83.5	68.0	89.2	66.1	94.7	89.4
Yes	22.5	40.3	32.1	15.1	16.5	32.0	10.8	33.9	.3	10.6
Provider’s gender (%)										
Female	46.2	54.6	85.5	19.9	25.9	24.4	7.6	19.4	26.8	9.0
Male	53.8	45.4	14.5	80.1	74.1	75.6	92.4	81.5	73.3	91.0
Provider’s age (years, %)										
<30	14.5	8.0	5.9	21.3	21.7	0.4	5.4	0.4	0.0	5.5
30–34	11.6	17.9	10.4	19.6	17.4	4.8	5.2	4.7	0.5	5.1
35–39	15.7	14.4	18.7	17.7	16.2	12.0	18.6	.6	12.9	18.6
40–44	24.5	32.8	23.3	27.6	9.6	8.4	19.6	8.9	19.4	19.0
40- ≥45	33.7	27.0	41.7	13.7	25.2	74.5	51.3	77.4	77.5	51.9
Provider’s position (%)										
Western medicine physician	46.5	47.9	77.0	13.1	13.2	62.2	25.3	66.0	2.3	24.2
Traditional medicine physician	6.2	24.0	5.7	14.5	11.7	2.8	4.0	3.0	3.3	3.9
Mid-level provider^3^	13.8	8.2	12.3	11.3	17.9	4.8	3.1	4.2	29.7	4.3
Pharmacist/pharmacy worker	14.1	6.0	0.4	14.6	16.8	3.5	23.8	3.7	0.0	22.5
Community health worker^4^	19.4	13.9	4.7	46.6	40.4	26.7	43.8	23.1	64.8	45.2
Experience as health provider (years,										
<5	44.2	41.3	48.5	26.7	30.1	2.3	4.7	2.0	0.0	4.6
5–9	18.6	21.6	25.6	24.9	28.8	7.6	14.0	4.1	.7	14.5
10–14	29.1	26.6	25.3	29.7	27.1	15.6	20.1	16.3	30.9	20.0
≥15	8.2	10.5	0.6	18.7	13.9	74.5	61.2	77.6	65.4	60.9
Full-time worker (%)										
No	20.1	24.8	30.5	20.4	17.6	16.7	10.5	17.8	.6	10.3
Yes	79.9	75.2	69.5	79.6	82.4	83.3	89.5	82.3	92.4	89.7
Average weekly hours worked (%)										
<40	28.5	20.9	26.2	48.2	6.1	15.3	12.2	15.8	9.3	12.6
40–60	37.6	33.2	33.7	31.6	43.2	55.4	51.0	57.0	50.0	50.4
≥60	33.9	46.0	40.1	40.7	30.7	29.3	36.8	27.2	40.7	37.0

*Notes*: All data are weighted using Taylor’s linearization method. All differences between the two surveys are statistically significant at p-level<0.05 based on chi-squared tests.

^1^No actual provision of either medical or surgical abortion

^2^All pharmacies in India are private

^3^ Includes nurses, auxiliary nurse midwives, clinic coordinators, lab technicians, family planning counselors

^4^Includes registered medical practitioners and women medical practitioners.

Male relative to female providers had lower odds of offering surgical abortions (Table 2). Compared to physicians practicing western medicine, all other types of providers were less likely to offer surgical abortions and mid-level providers were less likely to offer medical abortions. Providers working ≤40 hours/week were less likely to offer abortion services than those working 41–60 hours/week. We also found important differences in key factors associated with abortion provision between 2004 and 2012/2013. In 2004, but not in 2012/2013, the likelihood of offering surgical abortion and any abortion services was significantly lower among public sector compared to private sector providers; male providers were significantly less likely than female providers to offer medical abortions or any abortion services; and compared to physicians practicing western medicine, community health workers were significantly less likely to offer medical abortion. Conversely, in 2012/2013, yet not in 2004, providers in Jharkhand were significantly more likely than those in Bihar to offer medical abortion; heads of facilities were 4 times significantly more likely to provide surgical abortions; providers <30 years of age were 3.7 times more likely to offer abortion services; compared to physicians practicing western medicine, mid-level providers were significantly less likely to offer any type of abortion services; more experienced providers (≥5 years vs <5 years) and those working more hours (>60 vs 41–60 hours/week) were significantly more likely to offer abortion services than their counterparts.

**Table 2:** Results from multivariate logistics regression models of medical, surgical, and any abortion service provision in Bihar and Jharkhand, India: 2004 and 2012/2013.

Provider characteristics	2004			2012/2013		
	Surgical abortion^1^	Medical abortion^1^	Any abortion service^1^	Surgical abortion^1^	Medical abortion^1^	Any abortion service^1^
	Adj-OR (95% CI)^2^			Adj-OR (95% CI)^2^		
Jharkhand State (Bihar State = ref)	1.50 (0.72, 3.11)	0.67 (0.28, 1.64)	0.89 (0.37, 2.16)	0.72 (0.40, 1.27)	**1.61 (1.12, 2.30)**	1.35 (0.66, 2.77)
Rural location (urban = ref)	0.44 (0.16, 1.20)	0.43 (0.13, 1.38)	0.95 (0.47, 1.93)	0.45 (0.20, 1.01)*	0.54 (0.22, 1.31)	0.61 (0.21, 1.76)
Facility sector (private = ref)						
Public	**0.04 (0.001, 0.91)**	omitted	**0.15 (0.06, 0.40)**	0.40 (0.07, 2.17)	0.94 (0.20, 4.35)	0.83 (0.20, 3.44)
Private pharmacy^3^	omitted	0.52 (0.05, 5.06)	0.53 (0.22, 1.26)	0.62 (0.05, 8.23)	.92 (0.08, 11.06)	0.67 (0.07, 6.55)
Head of health facility (no = ref)	0.28 (0.07, 1.06)*	0.79 (0.19, 3.33)	1.03 (0.17, 6.21)	**4.08 (1.48, 11.29)**	0.52 (0.25, 1.09)*	0.72 (0.38, 1.36)
Male provider (female = ref)	**0.18 (0.08, 0.38)**	**0.06 (0.02, 0.15)**	**0.59 (0.38, 0.91)**	**0.11 (0.03, 0.40)**	1.06 (0.18, 6.37)	0.56 (0.17, 1.81)
Provider’s age (years; 35–39 = ref)						
<30	0.93 ((0.22, 3.87)	0.66 (0.10, 4.44)	0.96 (0.71, 1.30)	9.47 (1.62, 55.47)	4.36 (1.17, 16.27)	**3.69 (1.03, 13.18)**
30–34	0.57 (0.21, 1.55)	1.46 (0.35, 6.11)	0.85 (0.42, 1.73)	3.32 (0.87, 12.67)	2.74 (0.69, 10.84)	2.68 (0.82, 8.84)*
40–44	0.79 (0.34, 1.80)	2.01 (0.73, 5.49)	.14 (0.98, 4.65)*	2.12 (0.36, 12.54)	1.29 (0.17, 9.99)	0.82 (0.12, 5.35)
≥45	0.55 (0.17, 1.76)	0.39 (0.09, 1.72)	1.16 (0.60, 2.24)	3.45 (0.74, 16.04)	0.63 (0.08, 4.78)	0.54 (0.08, 3.65)
Provider’s position (western med physician = ref)						
Traditional medicine physician	**0.06 (0.01, 0.29)**	1.25 (0.17, 9.23)	0.53 (0.09, 3.11)	**0.04 (0.01, 0.17)**	0.55 (0.10, 2.98)	0.43 (0.09, 2.09)
Mid-level provider^4^	**0.04 (0.01, 0.13)**	**0.04 (0.01, 0.36)**	0.43 (0.13, 1.43)	**0.06 (0.01, 0.41)**	**0.04 (0.004, 0.45)**	**0.06 (0.10, 0.33)**
Pharmacist/pharmacy worker	**0.14 (0.02, 0.82)**	0.19 (0.01, 2.76)	0.33 (0.07, 1.58)	**0.11 (0.02, 0.69)**	.96 (0.14, 27.23)	2.41 (0.19, 31.04)
Community health worker^5^	**0.01 (0.003, 0.05)**	**0.05 (0.004, 0.54**)	0.31 (0.08, 1.28)	**0.19 (0.09, 0.41)**	0.51 (0.14, 1.86)	1.25 (0.39, 4.03)
Experience as health provider (years;<5 = ref)						
5–9	**0.11 (0.03, 0.35)**	0.31 (0.06, 1.47)	0.55 (0.26, 1.16)	2.44 (0.48, 12.41)	2.31 (0.46, 11.68)	**4.96 (1.24, 19.85)**
10–14	0.35 (0.10, 1.17)*	0.31 (0.07, 1.39)	0.73 (0.37, 1.45)	3.13 (0.55, 17.77)	4.65 (0.94, 23.04)*	**6.95 (2.24, 21.50)**
≥15	**0.41 (0.18, 0.91)**	0.82 (0.22, 2.99)	0.68 (0.31, 1.48)	4.47 (0.72, 27.45)	**8.58 (1.04, 70.72)**	**14.08 (2.87, 69.06)**
Full-time health worker (no = ref)	2.22 (0.64, 7.72)	0.38 (0.07, 2.24)	0.90 (0.41, 1.98)	0.68 (0.15, 3.03)	0.65 (0.33, 1.28)	0.68 (0.31, 1.49)
Average weekly hours worked (41–60 = ref)						
≤40	1.18 (0.24, 5.80)	0.33 (0.03, 3.22)	**0.50 (0.31, 0.79)**	0.41 (0.14, 1.20)*	**0.41 (0.19, 0.89)**	**0.38 (0.18, 0.79)**
>60	1.69 (0.81, 3.53)	2.59 (0.59, 11.45)	1.10 (0.63, 1.93)	0.99 (0.44, 2.23)	1.19 (0.54, 2.58)	**2.14 (1.00, 4.58)**

Notes: All data are weighted using Taylor’s linearization method.

^1^Provision of any other reproductive health service used as comparison

^2^Models adjusted for all covariates shown in the table

^3^All pharmacies in India are private

^4^Includes nurses, auxiliary nurse midwives, clinic coordinators, lab technicians, family planning counselors

^5^Includes registered medical practitioners and women medical practitioners.

Figures in bold are statistically significant at p<0.05

*Figures are statistically significant at p<0.10.

Over 96% of providers interviewed in 2012/2013 cite the ease of delivery, safety and efficacy profiles of abortion medications, and the demand for the procedure as reasons for providing medical abortion to their patients (Table 3). Significantly fewer providers in 2012/2013 than in 2004 report offering medical abortions because they are more profitable than other abortion methods or because they give women more control over their abortion than surgical procedures. On the other hand, concerns regarding the procedure’s delivery, safety and efficacy were more frequently cited as reasons for not offering medical abortions in 2012/213 than in 2004. For example, in 2012/2013, one in five providers not offering medical abortions reported that governmental requirements for mifepristone provision were too complicated, and two in five providers had concerns about the safety and efficacy of the mifepristone-misoprostol combination. Only 45.5% and 21.3% of providers cited a lack of interest in medical abortion and their knowledge of medical abortion, respectively, as motivation for not providing this service in 2004 compared to almost 70% of those interviewed in 2012/2013.

**Table 3:** Abortion providers’ reasons to offer medical abortion in Bihar and Jharkhand, India: 2004 versus 2012/2013.

Reasons		% providers offering MA		% providers not offering MA	
		2004	2012/2013	2004	2012/2013
Market demand	There is demand for MA from patients	98.2	99.0	N/A	
	MA is more profitable than other abortion methods	**91.7**	**82.8**		
	Other providers in the area are offering MA services	67.4	69.1		
Ease of delivery	MA is easier than surgical abortion to provide to patients	**91.3**	**96.2**		
	MA is a non-invasive procedure	**83.8**	**99.6**		
Safety and efficacy of MA	Use of Mifepristone-Misoprostol is safe and effective	**82.7**	**98.4**		
Patients’ rights	MA gives women greater control over their abortion than surgical abortion	**97.7**	**77.4**		
Market demand	There is not much demand for MA from patients	N/A		**39.6**	**47.3**
	Surgical abortion generates more income			**34.3**	**4.1**
Ease of procedure delivery	I prefer to use other drugs/medications			**34.0**	**17.7**
	I prefer to use surgical abortion			**28.9**	**3.1**
	Mifepristone is too expensive			**6.6**	**23.1**
	Government requirements to provide Mifepristone are too complicated			**5.3**	**20.3**
	I prefer to use a regimen using Misoprostol alone			2.1	4.7
Safety and efficacy of MA	There is no surgical back-up available near my practice			**42.3**	**25.7**
	I have concerns about the efficacy of MA			**7.5**	**39.5**
	I have concerns about the safety of MA			**7.1**	**39.6**
Patients’ rights	I have concerns about women complying with MA regimen			**37.4**	**25.6**
Personal interest	I have no interest in performing abortion services			**45.5**	**68.7**
	I do not know enough about MA			**21.3**	**69.7**

Notes: All data are weighted using Taylor’s linearization method. Figures in bold indicate that differences between the two surveys are statistically significant at p-level<0.05 based on chi-squared tests. MA, medical abortion; N/A, not applicable.

## Discussion

We found important changes in abortion service provision in the two northern Indian states between 2004 and 2013. Most importantly, our study documents an important uptake of medical abortion during this period, in line with other recent studies conducted in these and other Indian states [1]. For example, Acharya et al [5] found that 61.1% of the providers interviewed in Bihar and 73.4% of those interviewed in Maharashtra in 2009/2010 offered medical abortions. Such finding is not surprising given studies showing that medical abortion is not only acceptable, but increasingly requested by Indian women [2, 13, 15–21]. Other factors contributing to the observed increase in medical abortion provision include: an increased awareness about its legality and on-going efforts to further expand its provision through MTP Act changes [5,6]; improved provider knowledge of its safety and efficacy profile as well as national clinical guidelines regarding its use up to 63 days’ gestation [2–4]; and higher availability of medical abortion drugs in public and private facilities and pharmacies as both misoprostol and mifepristone were included in the National List of Essential Medicines in 2011 [1].

As of 2011, 79% and 77% of facilities registered to provide abortion services in Bihar and Jharkhand, respectively, were in the private sector [1]. While our analysis confirms that private sector providers were more likely to offer abortion services than public sector providers in 2012/2013, we also find that a considerably higher proportion of public sector providers did so in 2012/2013 compared to 2004. This is likely in response to the Indian government’s efforts to expand access to safe abortion services. An abortion provider gender gap persisted in 2012/2013 only with regard to surgical abortions–male providers were significantly less likely to provide such procedures than female providers. This was expected given that Indian women tend not to seek health care if a female provider is not available [22].

Both in 2004 and 2012/2013, physicians practicing western medicine were significantly more likely than all other types of providers to offer surgical abortion procedures. In addition, we found that facility managers were significantly more likely to offer surgical abortions in 2012/2013 but not in 2004, possibly because they had more experience with this type of procedure or because no other provider in the facility could offer this service when demanded or needed by patients. However, our results show that other types of medical providers and community health workers have been offering surgical and medical abortion services in both states. Thus, it is important to recognize the need for increased awareness and knowledge of both surgical and medical abortion procedures among a large cadre of Indian providers, especially in light of the pending MPT Act amendments. A recent Cochrane systematic literature review [23] and studies conducted in India have shown promising results vis-à-vis provision of medical abortion by non-allopathic physicians and other types of providers. Failure rates following medical abortions performed by mid-level providers were low (5–6%), and those among nurses and ayurvedic physicians were statistically equivalent to those for allopathic physicians [24]. Also, pharmacists and community health workers, representing two thirds of medical abortion providers in the two states in 2012/2013, should be targeted with information (e.g. through media, professional organizations, supervisors) regarding the correct prescription of abortion medications, their side-effects profiles, and the need to refer patients seeking medical abortion to health facilities.

The demand for medical abortions may increase in India in future years, especially if a wider range of providers offer this service. On the supply side, the ease of delivery and the safety and efficacy profile of medical abortions became more important reasons for providers to offer this service or not in 2012/2013 compared to 2004. The consideration of patients’ rights weighed less in providers’ decision regarding provision of medical abortions. Conversely, the lack of a personal interest in this procedure influenced over two thirds of providers who were not offering medical abortion in 2012/2013, significantly more than in 2004. In light of these findings, future studies should aim to shed more light on providers’ motivation to offer medical abortions. While not all reasons for the rise of medical abortion provision are known or measurable, it is possible that son preference and sex-selective abortion play a role. Notably, male: female sex ratios at birth are considerably higher in Bihar (1.07) and Jharkhand (1.09) than in other Indian states [25]. Thus, sex-selective abortion should be considered by future studies examining reasons for changes in abortion practices over time.

Our study is not without limitations. First, in lieu of longitudinal data, we used two cross-sectional surveys with a standardized questionnaire; thus, findings should be interpreted with caution. We conducted a census of reproductive health providers in 2004, of which we randomly selected one per facility for this analysis, yet we interviewed only one provider in each facility for the 2012/2013 survey. Moreover, for the latter, the provider self-selected to be interviewed and, given cultural norms in India, more senior providers are over-represented in the 2012/2013 sample. We aimed to overcome this selection bias in our regression analyses by adjusting all models not only for providers’ age, but also for their experience, weekly hours worked, and serving as facility managers. Of note, the association between providers’ age and their being abortion providers (or not) was not statistically significant at p<0.05 (data not shown); thus, there is no indication of differential misclassification by age among abortion providers in the 2012/2013 sample.

This study documents changes in abortion provision in two Indian states over a 9-year period. More research is needed to understand the factors that led to these changes, assess potential future changes if current MTP Act amendments become law, and learn how to further improve Indian’s women access to safe abortion services. Data from developed countries show that both medical abortion and surgical abortions procedures are relatively safe [26]. Progress towards making abortions safe and abortion deaths rare in India will have an important impact on decreasing maternal mortality and morbidity in this country.

## Supporting information

S1 File India 2004 data file. (DTA)

S2 File India 2012 data file. (DTA)
